# Theranostics using ^89^Zr/^177^Lu-labeled antibody targeting erythropoietin-producing hepatocellular A2 (EphA2)

**DOI:** 10.1007/s00259-025-07139-9

**Published:** 2025-02-12

**Authors:** Tadashi Watabe, Takumi Iwasawa, Hiroyuki Kimura, Yoshifumi Shirakami, Sadahiro Naka, Kazuko Kaneda, Takanori Kobayashi, Marina Omokawa, Yusuke Yagi, Noriyuki Tomiyama, Kazunori Kato

**Affiliations:** 1 Department of Radiology, Graduate School of Medicine, Osaka University, Suita, Japan; 2 Institute for Radiation Sciences, Osaka University, Suita, Japan; 3 Institute of Life Innovation Studies, Toyo University, Tokyo, Japan; 4 Research Center for Experimental Modeling of Human Disease, Kanazawa University, Kanazawa, Japan; 5 Department of Pharmacy, Osaka University Hospital, Suita, Japan; 6 Forefront Research Center, Graduate School of Science, Osaka University, Toyonaka, Japan; 7 Faculty of Medicine, Dentistry and Pharmaceutical Sciences, Okayama University, Okayama, Japan; 8 Department of Radiological Technology, Faculty of Medicinal Science, Kyoto College of Medical Science, Nantan, Japan; 9 Faculty of Health and Sports Sciences, Toyo University, Tokyo, Japan

**Keywords:** EphA2, Lutetium (^177^Lu), Zirconium (^89^Zr), Theranostics, Antibody

## Abstract

**Purpose:**

Erythropoietin-producing hepatocellular A2 (EphA2) is abundantly expressed in various types of cancers. This study aimed to assess the therapeutic effect of ^177^Lu-labeled EphA2 antibody in combination with PET imaging using ^89^Zr-labeled antibody.

**Methods:**

An EphA2 monoclonal antibody (mAb) (clone 230-1) was labeled with ^89^Zr and ^177^Lu using DFO and NOTA chelators, respectively. HT-1080 (EphA2-expressing human fibrosarcoma) xenograft mice were intravenously administered [^89^Zr]Zr-EphA2 mAb (*n* = 6) for PET scanning, and [^177^Lu]Lu-EphA2 mAb (*n* = 12) to evaluate the biodistribution and specificity of uptake in HT-1080 xenografts in a blocking study. The antitumor effect was evaluated by administering 10 (*n* = 9) or 3MBq (*n* = 9) of [^177^Lu]Lu-EphA2 mAb, non-radiolabeled EphA2 mAb (*n* = 6), [^177^Lu]LuCl_3_ solution (10MBq, *n* = 6), or saline solution (control, *n* = 4) to HT-1080 xenograft mice. Body weight monitoring and preliminary blood tests were performed to evaluate toxicity. Immunohistochemical staining for EphA2 was performed using HT-1080 xenografts.

**Results:**

On [^89^Zr]Zr-EphA2 mAb PET, a markedly high accumulation was observed in the tumor xenograft 1 day after administration (SUVmax, 11.8 ± 4.98), which increased on day 5 (SUVmax, 20.1 ± 6.42). [^177^Lu]Lu-EphA2 mAb exhibited excellent uptake and retention in HT-1080 xenografts (49.4 ± 10.8%IA/g at 24 h and 101.8 ± 26.3%IA/g at 72 h after administration). Normal organs did not show high physiological uptake, except for the blood (16.9 ± 0.43%IA/g at 24 h and 16.1 ± 4.9%IA/g at 72 h). The blocking study demonstrated a significant reduction of tumor uptake 24 h after administration (47.2 ± 8.22 vs. 10.7 ± 0.52%IA/g, *P* = 0.02). Substantial tumor growth inhibition, and complete remission without severe toxicity (6 of 18 mice), was observed in HT-1080 xenograft mice after [^177^Lu]Lu-EphA2 mAb (3 and 10 MBq) administration. The non-radiolabeled EphA2 mAb showed a minor treatment effect compared with [^177^Lu]Lu-EphA2 mAb. Immunohistochemical staining revealed high EphA2 expression in the HT-1080 xenografts.

**Conclusion:**

[^89^Zr]Zr-/[^177^Lu]Lu-EphA2 mAb demonstrated high retention in tumors, and [^177^Lu]Lu-EphA2 mAb exhibited marked tumor shrinkage. EphA2 is a promising theranostics target for potential application in various types of cancer.

**Supplementary Information:**

The online version contains supplementary material available at 10.1007/s00259-025-07139-9.

## Introduction

Erythropoietin-producing hepatocellular A2 (EphA2) is a receptor tyrosine kinase and a member of the Eph receptor family, which exerts various physiological roles [1]. EphA2 is primarily involved in the embryonic development and maintenance of tissue homeostasis. Through interactions with the extracellular matrix, EphA2 regulates epithelial cell adhesion and morphogenesis, thereby preserving structural integrity and functional tissue formation. Additionally, EphA2 modulates signaling pathways involved in cell proliferation and survival and plays a critical role in the process of angiogenesis. Although it is highly expressed in proliferating epithelial cells, EphA2 expression is relatively low in most normal adult tissues [2]. In contrast, EphA2 is abundantly expressed in various types of cancers, including lung, esophageal, colorectal, cervical, ovarian, breast, prostate, and skin cancers [2]. Moreover, EphA2 expression is correlated with poor prognosis, increased metastatic potential, and reduced survival rates in patients with cancer [3]. Therefore, EphA2 has significant potential as both a valuable biomarker and a promising therapeutic target in cancer therapy.

Radioligand therapy using lutetium-177 (^177^Lu), which emits beta rays at 497 keV, has attracted substantial attention. [^177^Lu]Lu-DOTATATE (Lutathera^®^, Novartis) and [^177^Lu]Lu-PSMA-617 (Pluvicto^®^, Novartis) have been approved for neuroendocrine tumors and prostate cancer in the USA and Europe, respectively [4, 5]. These therapies have significantly extended progression-free and overall survival in refractory patients, highlighting the potential of radioligand therapy.

Compared with conventional low-molecular-weight compounds and peptides, antibodies are expected to exhibit enhanced selectivity and accumulation. Antibody–drug conjugates, which combine an antibody with a cytotoxic drug using an appropriate linker, are gaining attention because of their high therapeutic efficacy. In a phase 3 trial involving patients with HER2-low metastatic breast cancer, the use of a HER2-targeted antibody–drug conjugate (trastuzumab deruxtecan) resulted in significantly longer progression-free and overall survival than the physician’s choice of chemotherapy [6]. Currently, these techniques are being applied to radiotheranostics using antibodies labeled with diagnostic and therapeutic radionuclides, and hold great promise [7]. Currently, the application of radioligand therapy is limited to certain types of cancer. However, developing radiotheranostics that can be broadly applied as a pantumor treatment will be crucial in the future. EphA2-targeted theranostics represents one of the most promising approaches for achieving this goal.

In this study, we aimed to assess the diagnostic utility and therapeutic effect of an EphA2 monoclonal antibody (mAb) labeled with ^89^Zr or ^177^Lu ([^89^Zr]Zr-EphA2 mAb and [^177^Lu]Lu-EphA2 mAb, respectively) using a EphA2-expressing xenograft rodent model.

## Materials and methods

### Generation of mAb to human EphA2

EphA2 mAb (clone 230-1) was generated by immunizing BALB/c mice with human EphA2 proteins purified from the supernatants of HEK239-F transfectants expressing human EphA2, as previously reported [8]. The immunized splenocytes were then fused to P3 × 63Ag8U mouse myeloma cells. After cloning by limiting dilution twice, we selected a hybridoma cell colony termed 230-1 (mouse IgG1, κ). To confirm the reactivity of mAb 230-1 to EphA2, but not to other EphA and EphB families, EphA2 transfectants and recombinant proteins were examined using flow cytometry and enzyme-linked immunoassay.

## Deferoxamine (DFO)-linked EphA2 mAb

EphA2 mAb (clone 230-1) (500 µg) was diluted in 0.1 M NaHCO_3_ (pH 9.5, 25 µL), then added to a 12-fold molar excess of *p*-SCN-Bn-DFO (32 µg) diluted in 0.1 M NaHCO_3_ (pH 9.5, 125 µL), and mixed at room temperature for 24 h. The reaction mixture was purified by gel filtration chromatography using a PD-10 column (GE Healthcare), and the appropriate fractions were collected and concentrated using a centrifugal filter (Amicon Ultra 0.5 mL; Ultracel-100 K). The reaction progress was observed using matrix-assisted laser desorption-ionization time-of-flight mass spectrometry (MALDI-TOF-MS) (Bruker and Shimadzu Corporation). 3,5-Dimethoxy-4-hydrocinnamic acid and α-cyano-4-hydroxycinnamic acid were saturated with 0.1% trifluoroacetic acid solution/acetonitrile (1:1), respectively. The EphA2 mAb (clone 230-1) was dissolved in this aqueous solution and measured.

## Synthesis of [^89^Zr]Zr-EphA2 mAb

^89^Zr was produced via the nuclear reaction of ^89^Y(*p*, *n*)^89^Zr using a medical cyclotron, CYPRIS HM-18 (Sumitomo Heavy Industries), fitted with an yttrium (^89^Y) foil, measuring 10 × 11 mm and with a thickness of 0.15 mm, into a gold disk as the target material. After irradiation, the ^89^Y foil was dissolved in 5 mL of 6 M HCl and passed through a hydroxamate-based resin (Zr resin; Triskem) preconditioned with 2.5 mL of 2 M HCl to purify ^89^Zr. After washing with 10 mL of 2 M HCl and 10 mL of water for injection, ^89^Zr was eluted from the Zr resin using 1 M oxalic acid.

[^89^Zr]Zr-EphA2 mAb (clone 230-1) was prepared by labeling a DFO-linked EphA2 mAb with ^89^Zr. Briefly, 200 µL of 1 M 4-(2-hydroxyethyl)piperazine-1-ethane-sulfonic acid (HEPES) buffer (pH 6.7) and 65 µL of 2 M Na_2_CO_3_ were added to 200 µL of ^89^Zr-oxalic acid (~ 80 MBq). Thereafter, 200 µL of EphA2 mAb coupled with the DFO chelate (497 µg) was dissolved in 0.5 M HEPES buffer (pH 6.7), reacted at 27 °C, and centrifuged at 550 rpm for 60 min. The [^89^Zr]Zr-EphA2 mAb was obtained with more than 95% radiochemical purity via radio-thin-layer chromatography (miniGITA; Raytest) analysis using iTLC-SG paper and 20 mM citric acid buffer (pH 5.0) after purifying the reaction mixture using a PD-10 column (Cytiva, Tokyo, Japan) and 0.25 M sodium acetate buffer (pH 5). The final solutions of the products contained 23.1 MBq/mL activity and 132 µg/mL antibody in 0.25 M sodium acetate buffer (pH 5). An average of 5.3 DFO chelate was coupled to an anti-EphA2 mAb, which was measured using MALDI-TOF.

### 1,4,7-triazacyclononane-*N, N’,N"*-triacetic acid (NOTA)-linked EphA2 mAb

EphA2 mAb (clone 230-1) (500 µg) was diluted in 0.1 M NaHCO_3_ (pH 9.5, 25 µL), added to a 20-fold molar excess of *p*-SCN-Bn-NOTA (38 µg) diluted in 0.1 M NaHCO_3_ (pH 9.5, 150 µL), and mixed at room temperature for 24 h. The reaction mixture was purified by gel filtration chromatography using a PD-10 column (GE Healthcare), and the appropriate fractions were collected and concentrated using a centrifugal filter (Amicon Ultra 0.5 mL, Ultracel-100 K; Merck). The reaction progress was observed using MALDI-TOF-MS (Bruker and Shimadzu Corporation). 3,5-Dimethoxy-4-hydrocinnamic acid and α-cyano-4-hydroxycinnamic acid were saturated with 0.1% trifluoroacetic acid solution/acetonitrile (1:1), respectively. EphA2 mAb was dissolved in this aqueous solution and measured.

### Synthesis of [^177^Lu]EphA2 mAb

[^177^Lu]LuCl_3_ (lutetium chloride dissolved in 0.04 M HCl) was purchased from POLATOM and Eckert & Ziegler Radiopharm. Representatively, an EphA2 mAb (clone 230-1) coupled with NOTA chelates (0.5 mg) was dissolved in 0.3 mol/L sodium acetate (pH 6.5) and reacted with a [^177^Lu]LuCl_3_ solution (100 MBq) at 44 °C for 15 min. Then, the solution was added to a solution of 10% sodium ascorbate and reacted at 44 °C for 45 min. The obtained [^177^Lu]Lu-EphA2 mAb was analyzed by electrophoresis (strip, cellulose acetate; constant voltage,133 V; constant current, 1 mA/cm; eluant, 0.06 mol/L barbital buffer; pH 8.5; duration, 30 min) as a commonly used and validated method. Activity on the strip was measured using a Typhon FLA7000 bioimager (Cytiva). The radiochemical purity of the products ranged from 92.0 to 96.1% (average, 94.7%), and remained above 90% at 24 h after preparation. Concentrations of activity of the products were adjusted to 10 MBq/mL and 3 MBq/mL for treatment and biodistribution evaluation, respectively. Final solutions of the products contained 500 µg/mL antibody and 1% sodium ascorbate in 0.1 mol/L sodium acetate buffer solutions (pH 6.5). An average of 6.1 NOTA chelates was coupled to the EphA2 mAb, which was measured using MALDI-TOF.

### In-vitro analysis using HT-1080 WT and EphA2-KO cells

Target sequence identification for designing gRNA was performed using the online software CRISPRdirect (https://crispr.dbcls.jp/). The gRNA sequence GAGGGGCAGAAGTTGCGCGC (AGG) in exon 1 of EphA2 was cloned into a pCas-Guide-EF1a-GFP vector (OriGene Technologies). HT-1080 cells (EphA2-expressing human fibrosarcoma cells) were obtained from American Type Culture Collection. Cells were transfected with the CRISPR Cas9 plasmid using the Neon transfection system (Thermo Fisher Scientific). At 48 h post-transfection, the cells were trypsinized and sorted via fluorescence-activated cell sorting (FACS) to obtain green fluorescent protein–positive cells using FACSMelody (Becton Dickinson). Clonal selection of single green fluorescent protein–positive cells yielded several viable clones, which were screened for *EphA2* gene knockouts (KO) by Sanger sequencing. EphA2 protein levels were then compared with those in wild-type (WT) cells by western blotting.

The reactivity of EphA2 mAb (clone 230-1) and NOTA–linked EphA2 mAb (clone 230-1) to EphA2 on cancer cell surfaces was determined using flow cytometry. Approximately 1 × 10^5^ HT-1080 WT or HT-1080 EphA2-KO cells were incubated with various concentrations (31.25–2,000 ng/mL) of mAb in 20 µL phosphate-buffered saline (PBS; pH 7.4) with 2% fetal bovine serum (FBS; staining buffer) for 60 min on ice. The cells were then washed and stained with the phycoerythrin-conjugated goat anti-mouse IgG secondary antibody (Invitrogen) for 30 min on ice. The cell suspension was washed three times with PBS and analyzed using a FACSymphony A1 flow cytometer (BD Immunocytometry Systems).

### Preparation of xenograft models

HT-1080 cells were maintained in a culture medium (RPMI 1640 with l-glutamine and phenol red; Fujifilm Wako Pure Chemical) with 10% heat-inactivated FBS and 1% penicillin-streptomycin. Male nude mice (BALB/cSLC-nu/nu, origin: the Institute of Medical Science, the University of Tokyo, total *n* = 46) were purchased from Japan SLC, Inc. (Hamamatsu, Japan). The mice were housed under a 12 h light/12 h dark cycle with free access to food and water and were allowed to acclimate for at least one week. The housing chamber temperature was maintained at approximately 23 °C. Tumor xenograft models were established by the subcutaneous injection of 1 × 10^6^ HT-1080 cells suspended in 0.1 mL PBS in nude mice. The mice were evaluated 2 weeks after implantation, when the tumor size reached approximately 1 cm in diameter. The protocol was approved by the Animal Care and Use Committee of the Osaka University Graduate School of Medicine (approval number: 30-088-009). Euthanasia was performed under deep anesthesia using isoflurane inhalation when signs of intolerable suffering or a significant decrease in body weight (reduction of more than 30% compared to baseline) were observed. A tumor diameter of more than 2 cm (approximately 4,000 mm³) was also defined as the humane endpoint.

### [^89^Zr]Zr-EphA2 mAb PET imaging and analysis

[^89^Zr]Zr-EphA2 mAb (clone 230-1) (1.30 ± 0.09 MBq, approximately 7.62 ± 0.52 µg of EphA2 mAb, adjusted to a volume of 0.1 mL) was intravenously administered to HT-1080 xenograft mice (9 weeks old, body weight = 23.4 ± 0.9 g, *n* = 6). Static PET/CT scanning was performed 1 and 5 days after administration, by PET scan durations of 10 and 20 min, respectively, under isoflurane anesthesia using a small-animal PET scanner (Siemens Inveon PET/CT). After the PET scan on day 5, the mice were euthanized, and the activity and weight of the major organs were determined using a well counter (BeWell; Molecular Imaging Laboratory). All PET data were reconstructed using 3-dimensional ordered-subset expectation maximization (16 subsets, 2 iterations), followed by maximum a posteriori (OSEM3D-MAP) with scatter and attenuation correction. The regional uptake of activity was decay-corrected for the injection time and expressed as a standardized uptake value (SUV). Regions of interest (ROIs) were manually defined on the tumor (ellipsoid-sphere ROI adjusted to the tumor size) and the muscle tissue (3 mm diameter spherical ROI in the right forelimb), referencing anatomical CT images using Osirix MD version 14.0 (Pixmeo SARL).

### Evaluation of biodistribution of [^177^Lu]Lu-EphA2 mAb

HT-1080 xenograft mice (*n* = 6) were intravenously administered [^177^Lu]Lu-EphA2 mAb (clone 230-1) (3.12 ± 0.21 MBq, 50 µg) to evaluate biodistribution under isoflurane anesthesia. The volume of each injected solution was adjusted to 0.1 mL (as for the following experiments). At 24 and 72 h after administration, the mice were euthanized, and the tumors and major organs were collected. The activity and weight of the samples were measured using a well counter system. A blocking study was also performed by pre-administration of non-radiolabeled EphA2 mAb (500 µg) (5 min before the administration of [^177^Lu]Lu-EphA2 mAb (2.93 ±0.13 MBq)) to evaluate the uptake specificity of [^177^Lu]Lu-EphA2 in HT-1080 xenograft (*n* = 6). Uptake levels were compared 24 h post-administration between the blocking and non-blocking groups. The absorbed dose (Gy) in the tumor was calculated based on the S value (0.0233 mGy/MBq·s) reported in a previous publication [9]. The residence time (s) was determined using the trapezoidal method with biodistribution data collected at 24 and 72 h, assuming only physical decay occurs after 72 h.

### Radioimmunotherapy using [^177^Lu]Lu-EphA2 mAb

The antitumor effect was evaluated by administering 10 MBq (9.7 ± 0.78 MBq, 46–150 µg, *n* = 9) or 3 MBq (2.84 ± 0.45 MBq, 12–50 µg, *n* = 9) of [^177^Lu]EphA2 mAb (clone 230-1) (10.36 ± 0.54 MBq), non-radiolabeled EphA2 mAb (clone 230-1) (50 µg, *n* = 6), [^177^Lu]LuCl_3_ solution (10 MBq, *n* = 6), or saline (control, *n* = 4) in HT-1080 xenograft mice (8 weeks old; body weight, 22.1 ± 1.24 g). Tumor size and body weight were monitored to evaluate treatment effects and systemic side effects. The tumor volume (mm^3^) was measured with a caliper and calculated using the elliptical sphere model equation. Preliminary blood tests were performed to evaluate hematological and kidney toxicity using iSTAT (Abbott).

### Immunohistochemistry

Immunohistochemical staining was performed to confirm EphA2 expression in the tumor xenografts using an anti-EphA2 antibody. After the animals were euthanized, tumor xenografts were resected and fixed with 4% paraformaldehyde (overnight, 4 °C). The fixed tissues were immersed in 30% sucrose in PBS (overnight, 4 °C). The frozen sections (slice thickness: 10 μm) were incubated with an anti-EphA2(D4A2) rabbit mAb (CST #6997; Cell Signaling Technology, 100-fold dilution). Immunohistochemistry was performed using Dako EnVision + System– HRP Labeled Polymer Anti-Rabbit (K4003) (DAKO Corp.). The stained sections were analyzed by microscopy (Keyence BZ-9000). A total of five mice were used, with one section prepared per mouse. Two locations were selected per section, and each location was captured at four magnifications: ×40, ×100, ×200, and ×400.

### Statistical analyses

Comparisons between the two groups were performed using the Mann–Whitney U test for tumor size and body weight, and the *t*-test, following normality assessment with the Shapiro-Wilk test, for flow cytometry analysis and blocking experiments. Statistical analyses were performed using SPSS version 25.0 (IBM Corp.), and differences were considered statistically significant at a *p*-value of < 0.05.

## Results

**Fig. 1:**
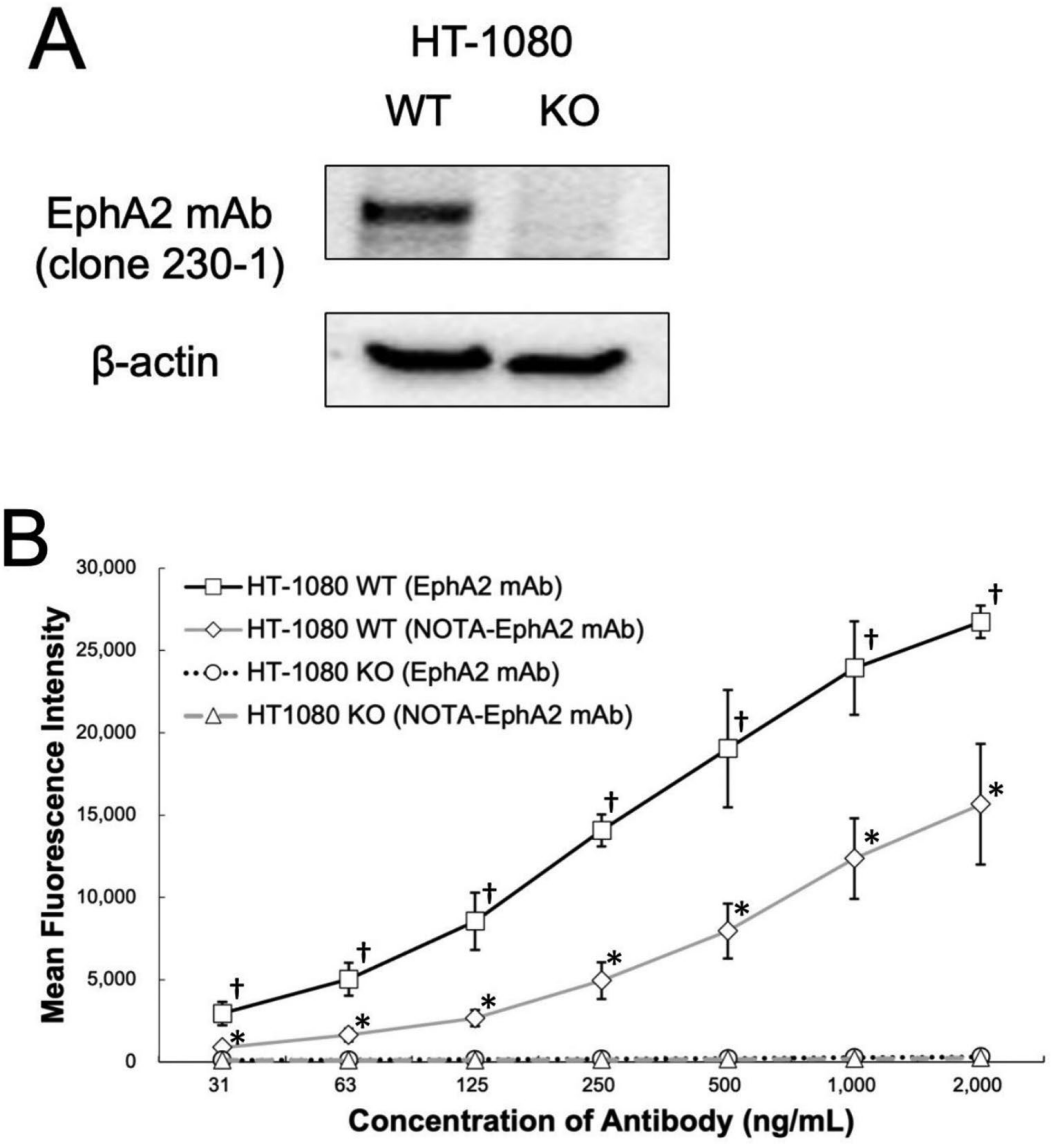
Results of western blotting analysis for EphA2 in HT-1080 WT and HT-1080 EphA2-KO cells. The full uncropped images of gels and blots are provided in the supplementary Fig. 1. (**B**) Results of flow cytometry analysis to evaluate the affinity of EphA2 mAb (clone 230-1) with and without NOTA chelator to HT-1080 WT and HT-1080 EphA2-KO cells. (†, *p* < 0.05 comparing HT-1080 WT and KO groups for EphA2 mAb, *, *p* < 0.05 comparing HT-1080 WT and KO groups for NOTA-EphA2 mAb)

The results of the western blotting analysis of EphA2 in HT-1080 WT and HT-1080 EphA2-KO cells are shown in Fig. 1A. A strong intensity band was detected in the WT, whereas no signal was detected in the KO cells, indicating the successful knockdown of EphA2 expression in HT-1080 EphA2 KO cells. In vitro analysis showed that the mean fluorescence intensity was significantly higher in HT-1080 WT cells for both EphA2 mAb and NOTA-linked EphA2 mAb than in HT-1080 EphA2-KO cells (Fig. 1B), suggesting the specific binding of EphA2 mAb to the EphA2 receptor in HT-1080 cells.

**Fig. 2:**
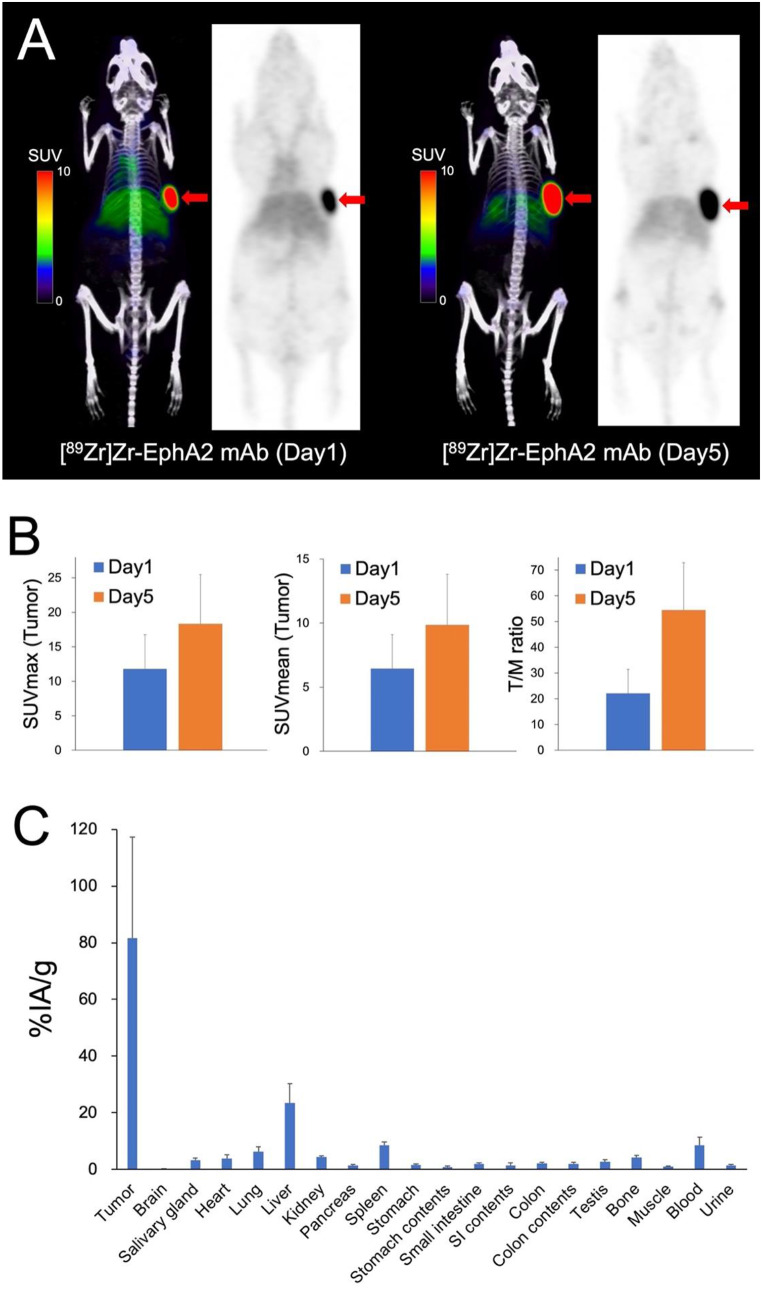
(**A**) [^89^Zr]Zr-EphA2 mAb (clone 230-1) PET images in HT-1080 xenograft mice at days 1 and 5 post-administration (arrows indicate tumor xenografts). (**B**) Quantitative analyses of tumoral uptake on [^89^Zr]Zr-EphA2 mAb (clone 230-1) PET. The T/M ratio is calculated as the ratio of tumor SUVmax to muscle SUVmean. (**C**) Biodistribution of [^89^Zr]Zr-EphA2 mAb (clone 230-1) PET at day 5 post-administration (tumor-to-blood ratio: 9.4 ± 2.4). (SI, small intestines)

The [^89^Zr]Zr-EphA2 mAb PET images of HT-1080 xenograft mice are shown in Fig. 2A. A markedly high accumulation of [^89^Zr]Zr-EphA2 mAb was observed in the tumor xenograft 1 day after administration (SUVmax, 11.8 ± 4.98), and it increased on day 5 (SUVmax, 20.1 ± 6.42) (Fig. 2B). A biodistribution study also revealed high accumulation in HT-1080 xenografts and mild physiological accumulation in the liver and spleen (Fig. 2C).

**Fig. 3:**
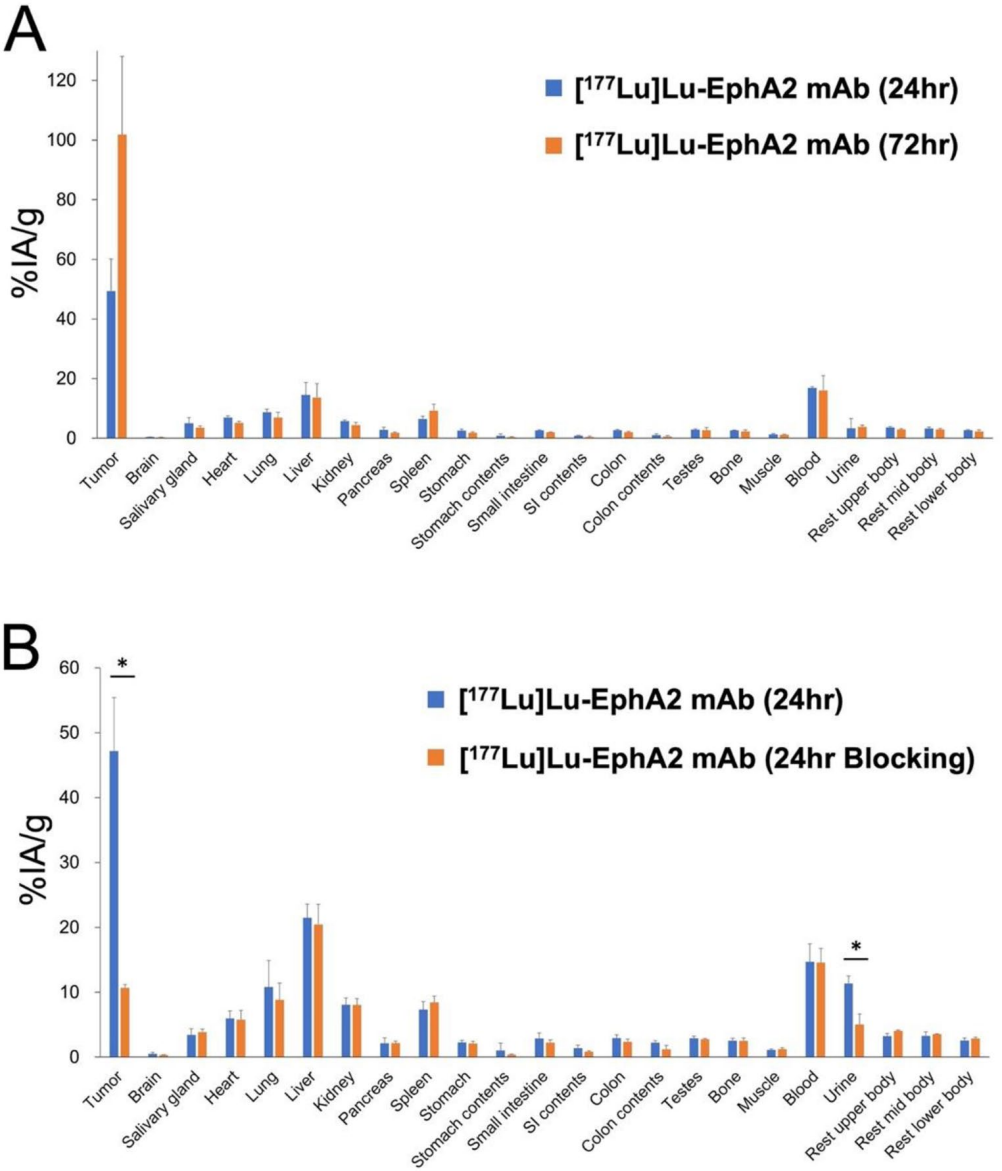
(**A**) Biodistribution of [^177^Lu]Lu-EphA2 mAb (clone 230-1) at 24 and 72 h post-administration in HT-1080 xenograft mice (mean ± standard deviation) (tumor-to-blood ratio: 2.92 ± 0.59 at 24 h and 6.81 ± 2.77 at 72 h). (**B**) Blocking study of [^177^Lu]Lu-EphA2 mAb (clone 230-1) at 24 h post-administration with and without pre-administration of non-radiolabeled EphA2 mAb. (tumor-to-blood ratio: 3.25 ± 0.52 at 24 h and 0.74 ± 0.10 at 24 h with blocking) (*p < 0.05, compared with the blocking group. SI, small intestines)

The biodistribution of [^177^Lu]Lu-EphA2 mAb is shown in Fig. 3A and showed high uptake and retention in the HT-1080 xenograft (49.4 ± 10.8%IA/g at 24 h and 101.8 ± 26.3%IA/g at 72 h after administration). In normal organs, high physiological uptake was not observed except for blood pooling (16.9 ± 0.43%IA/g at 24 h and 16.1 ± 4.9%IA/g at 72 h). The blocking study demonstrated significant reduction of tumor uptake at 24 h after administration (47.2 ± 8.22 vs. 10.7 ± 0.52%IA/g, *P* = 0.02) (Fig. 3B). It was suggested that its specific uptake was through EphA2 in the HT-1080 xenograft tumor.

**Fig. 4:**
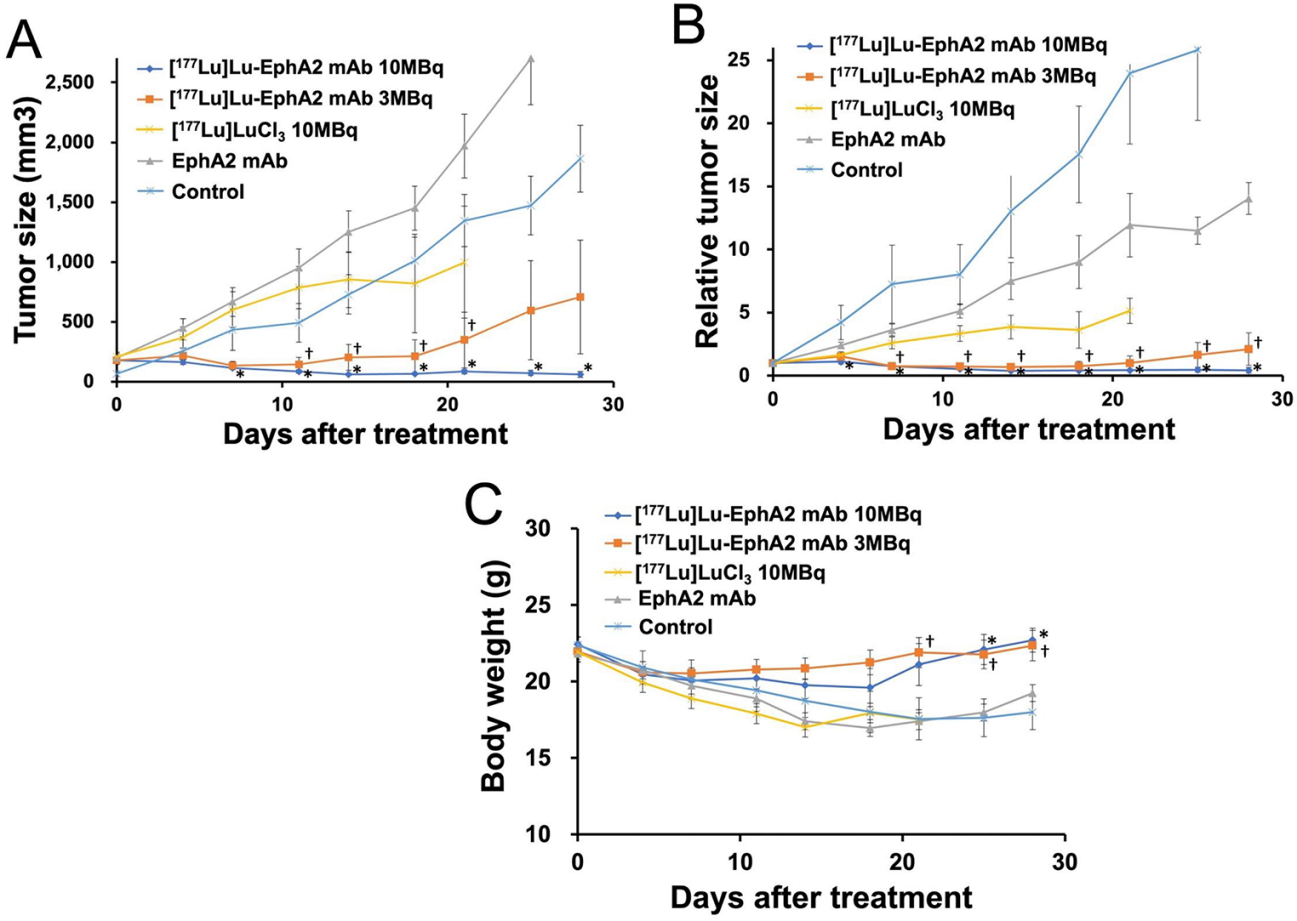
(**A**) Actual tumor growth curves and (**B**) relative tumor growth curves following the administration of [^177^Lu]Lu-EphA2 mAb (clone 230-1) (3 and 10 MBq), non-radiolabeled EphA2 mAb, [^177^Lu]LuCl_3_ solution, and control (saline) in HT-1080 xenograft mice (mean ± standard error). (**C**) Changes in body weight after drug administration. (†, *p* < 0.05 comparing [^177^Lu]Lu-EphA2 mAb 3 MBq and control groups; *, *p* < 0.05 comparing [^177^Lu]Lu-EphA2 mAb 10 MBq and control groups)

In the therapy experiment, substantial tumor growth inhibition was observed in the HT-1080 xenograft mice after the administration of [^177^Lu]EphA2 mAb (3 and 10 MBq) (Fig. 4A and B). Six of 18 mice showed complete tumor remission. The estimated absorbed doses in the tumor were 51.7 Gy for 3MBq and 172.4 Gy for 10MBq of [^177^Lu]Lu-EphA2 mAb. Even with a dose of 3MBq, a sufficient absorbed dose was delivered to the tumor. The non-radiolabeled EphA2 mAb showed a minor treatment effect compared with [^177^Lu]Lu-EphA2 mAb. The [^177^Lu]LuCl_3_ solution group showed some tumor growth inhibition, which was possibly due to perfusion or enhanced permeability. However, half of the mice died within 2 weeks after the administration of [^177^Lu]LuCl_3_ solution (10 MBq), indicating that appropriate targeting is essential.

**Fig. 5:**
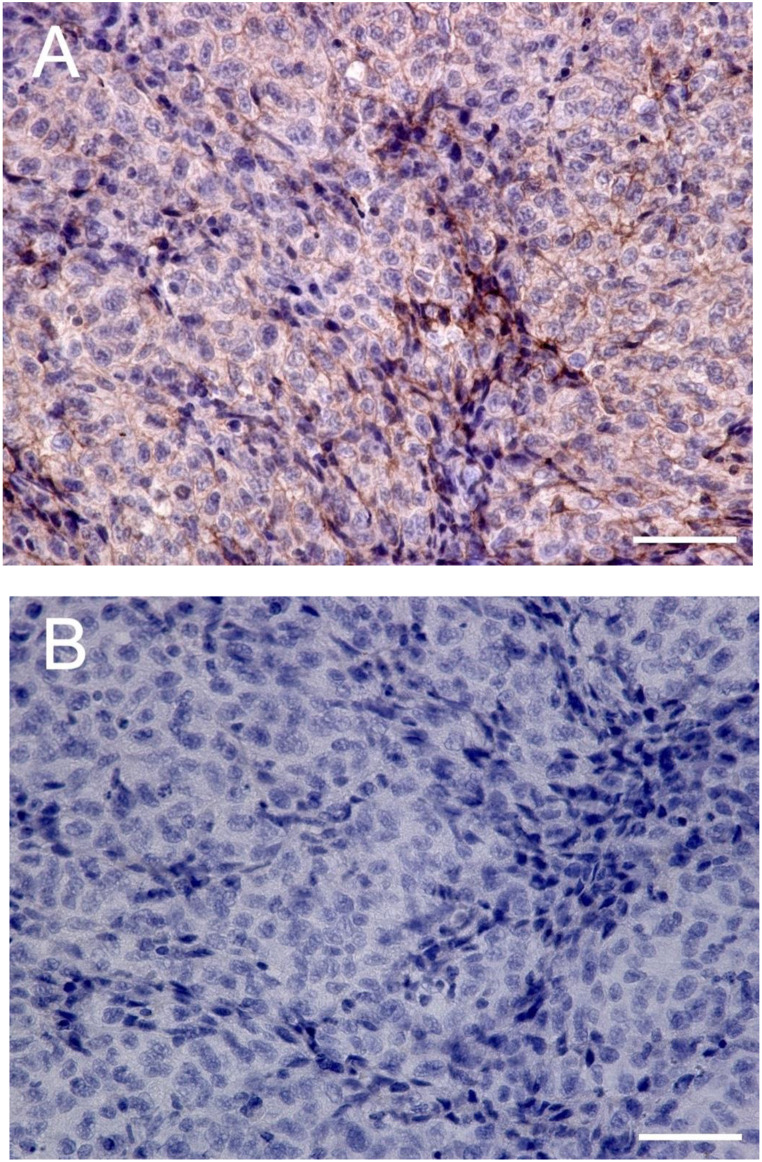
(**A**) EphA2 immunostaining in a HT-1080 xenograft (high magnification, ×400). (**B**) Negative controls of the corresponding sections in the absence of primary anti-EphA2 antibody. White bars indicate 50 μm

Regarding side effects, the group that was administered [^177^Lu]Lu-EphA2 mAb had no sustained weight loss; rather, the group was observed to have a weight increase, which was contrary to that observed in the control group, which exhibited cachexia due to tumor growth (Fig. 4C). Preliminary blood tests that were performed 6–7 weeks after administration showed no apparent renal or hematological toxicity in the [^177^Lu]Lu-EphA2 mAb group (Supplementary Table 1). Immunohistochemical staining revealed high expression of EphA2 in the HT-1080 xenografts (Fig. 5).

## Discussion

The present study demonstrated a significant therapeutic effect of the [^177^Lu]Lu-EphA2 mAb (clone 230-1) in HT-1080 xenografts, along with a markedly high accumulation in [^89^Zr]Zr-EphA2 mAb (clone 230-1) PET. The [^177^Lu]Lu-EphA2 mAb exhibited excellent tumor retention (101.8 ± 26.3%IA/g at 72 h post-administration) without substantial uptake by normal organs. Notably, a subset of mice (6/18) treated with 3 or 10 MBq [^177^Lu]Lu-EphA2 mAb achieved complete remission, which was attributed to the high absorbed dose delivered to the tumor (51.7 Gy for 3 MBq and 172.4 Gy for 10 MBq administration).

We selected ^89^Zr and ^177^Lu because their physical half-lives align well with the biological half-life of the antibody, and both are prominent PET/β-emitting radionuclides commonly used in clinical applications. For labeling with ^89^Zr, DFO was employed as the standard linker, a method confirmed in our previous studies to be both stable and reliable. For ^177^Lu, we selected NOTA as the chelator due to its ability to provide higher radiolabeling yields under mild conditions compared to DOTA.

A humanized EphA2 mAb engineered to enhance antibody-dependent cellular cytotoxicity (DS-8895a) was investigated in a phase 1 clinical trial in patients with advanced solid tumors [10]. The administration of DS-8895a at a dose of 20 mg/kg via intravenous infusion every 2 weeks was generally well tolerated in a cohort of 21 patients. However, only one patient demonstrated partial remission, suggesting limited efficacy as a monotherapy. Burvenich et al. evaluated ^111^In- and ^89^Zr-labeled EphA2 mAbs (DS-8895a) in breast cancer xenograft models (MDA-MB-231). Biodistribution studies and SPECT/PET imaging revealed significant uptake in EphA2-expressing xenografts, with minimal uptake in normal tissues [11]. Furukawa et al. developed a novel SPECT probe labeled with ^123^I targeting EphA2 and demonstrated its tumor uptake in a U87MG xenograft model [12]. We also demonstrated high accumulation of [^89^Zr]Zr-EphA2 mAb on PET in HT-1080 xenograft models. These findings underscore the potential feasibility of future clinical applications of the [^177^Lu]Lu-EphA2 mAb, especially in combination with companion diagnostics such as EphA2-targeting PET or SPECT.

A recent study reported that an EphA2-targeting bicycle peptide labeled with ^68^Ga, ^177^Lu, and ^111^In exhibited rapid and high accumulation in HT-1080 xenograft models [13]. However, the peptide demonstrated short tumor retention, with approximately 90% washout between 1 h and 24 h after administration, and no therapeutic outcomes were reported. Furthermore, persistently high accumulation was observed in the kidneys, raising concerns regarding potential renal toxicity. In contrast, our study demonstrated that EphA2-targeted radioligand therapy was effective in HT-1080 xenograft mice administered with [^177^Lu]Lu-EphA2 mAb, resulting in complete remission in some cohorts (6 of 18 mice) without severe toxicity. The sustained high accumulation of [^177^Lu]Lu-EphA2 mAb in tumors appears to be optimal for achieving a robust therapeutic effect.

We used HT-1080 human fibrosarcoma cells as a representative model for the theranostic evaluation, as reported in a previous study targeting EphA2 [13, 14]. HT-1080 cells are a suitable model system for the robust evaluation of therapeutic effects using [^177^Lu]Lu-EphA2 mAb due to their rapid growth, reaching an appropriate size for assessment approximately 2 weeks after implantation, with good reproducibility. Although the breast cancer cell line MDA-MB-231 has also been reported to exhibit high EphA2 expression, its relatively slow growth and variability in xenograft model formation make it less suitable for the initial evaluation.

EphA2 is abundantly expressed in a variety of cancers, including major types, such as lung, esophageal, colorectal, cervical, ovarian, breast, and prostate cancers, as well as rare ones, such as glioma, sarcoma, uveal melanoma, and malignant pleural mesothelioma [2, 15–18]. Thus, EphA2 is a promising pan-tumor target for radioligand therapy. Although fibroblast activation protein (FAP) is also a pan-tumor target, its expression is primarily observed in cancer-associated fibroblasts [19, 20]. A dual-target strategy that combines FAP and EphA2 may offer greater therapeutic potential. Furthermore, the strong internalization property of EphA2 mAb was reported in a previous study using [^111^In]In-BnDTPA-EphA2 mAb [21]. This makes EphA2 mAb an excellent candidate for targeted alpha therapy, as demonstrated in our previous study using an anti-Glypican-1 mAb labeled with the alpha emitter astatine (^211^At) [7].

When comparing the biodistribution between [^89^Zr]Zr-EphA2 mAb and [^177^Lu]Lu-EphA2 mAb, both exhibited very similar distribution, with substantial uptake in the tumor and mild physiological accumulation in the liver and spleen. Although antibody humanization and safety confirmation are necessary, [^89^Zr]Zr-EphA2 mAb PET can be utilized for the patient selection and dosimetric evaluation as a theranostic approach for [^177^Lu]Lu-EphA2 mAb therapy.

Regarding potential toxicity, EphA2 is expressed at low levels in most normal adult tissues, with some expression observed in certain epithelial cells [1, 2]. Since epithelial cells undergo constant maintenance and regeneration, long-term toxicity is not a major concern. However, a potential risk of toxicity is possible due to accumulation of the radioligand in organs such as the liver and kidneys, as well as potential for toxic effects on the bone marrow, which is highly sensitive to radiation.

In this study, the stability of radiochemical purities in the serum was not confirmed. According to a previous report, free ^177^Lu released in the body is known to accumulate in bone [22]. However, in our study, the accumulation of [^177^Lu]Lu-EphA2 antibody in bone was low, suggesting that stability was not a significant issue. We plan to conduct a more detailed analysis of blood samples in future studies. Accumulation in the liver and spleen is often observed with immuno-PET using antibodies, and this is considered a common physiological phenomenon. With regard to blood concentrations, antibodies generally have a long half-life in the bloodstream and we considered the observed levels were within the expected range. Furthermore, the relationship between antibody dose and tumor accumulation remains a subject for future investigation.

This study has some limitations. First, we used only a single xenograft model, HT-1080, in this study; thus, further evaluation, including survival analysis, across additional tumor models is needed. Second, the potential side effects of [^177^Lu]Lu-EphA2 mAb were not comprehensively assessed. Future studies should include detailed evaluations, such as hematological effects on blood cell counts over time and histological analyses of normal organs, to better understand their safety profiles. A major concern with the use of antibodies in radioligand therapy is hematotoxicity owing to their long retention times in the bloodstream. To address this, reducing the size of the antibodies, such as by switching to antibody fragments or nanobodies, may be necessary.

## Conclusion

[^89^Zr]Zr-/[^177^Lu]Lu-EphA2 mAb (clone 230-1) demonstrated high retention in tumors, and [^177^Lu]Lu-EphA2 mAb (clone 230-1) exhibited excellent tumor shrinkage. EphA2 is a promising target with potential for radioimmunotherapy in various cancer types. Further studies are warranted to confirm their efficacy and safety for clinical applications.

## Electronic supplementary material

Below is the link to the electronic supplementary material.

Supplementary Material 1

Supplementary Material 2

## Data Availability

Data is available upon request.
