# Supplementary material for: Theranostics using 89Zr/177Lu-labeled antibody targeting erythropoietin-producing hepatocellular A2 (EphA2)
Source: Eur J Nucl Med Mol Imaging. 2025 Feb 12;52(8):2887–97. doi: 10.1007/s00259-025-07139-9 (PMC12162727; doi:10.1007/s00259-025-07139-9)
Supplement: Supplementary file 1 — Supplementary Material 1 [file 259_2025_7139_MOESM1_ESM.docx]

**Supplementary Table 1.** Results of blood tests 6–7 weeks after the administration of [^177^Lu]Lu-EphA2 mAb (clone 230-1) (3 MBq and 10 MBq) in HT-1080 mice

|  | Na (mmol/L) | K (mmol/L) | Cl (mmol/L) | BUN (mg/dL) | Cre (mg/dL) | Hb (g/dL) | Ht (%) |
| --- | --- | --- | --- | --- | --- | --- | --- |
| Control | 149 | 6.2 | 115 | 38 | <0.2 | 9.5 | 28 |
|  | 126 | 4.9 | 103 | 29 | <0.2 | 8.8 | 26 |
| [^177^Lu]Lu-EphA2 mAb (3MBq) | 146 | 3.0 | 111 | 29 | <0.2 | 12.9 | 38 |
|  | 146 | 3.3 | 112 | 27 | <0.2 | 12.9 | 38 |
|  | 145 | 3.4 | 117 | 23 | <0.2 | 12.9 | 38 |
| [^177^Lu]Lu-EphA2 mAb (10MBq) | 143 | 5.6 | 112 | 28 | <0.2 | 12.9 | 38 |
|  | 145 | 4.3 | 113 | 20 | <0.2 | 13.3 | 39 |
