# Supplementary material for: Theranostics using 89Zr/177Lu-labeled antibody targeting erythropoietin-producing hepatocellular A2 (EphA2)
Source: Eur J Nucl Med Mol Imaging. 2025 Feb 12;52(8):2887–97. doi: 10.1007/s00259-025-07139-9 (PMC12162727; doi:10.1007/s00259-025-07139-9)
Supplement: Supplementary file 2 — Supplementary Material 2 [file 259_2025_7139_MOESM2_ESM.docx]

**
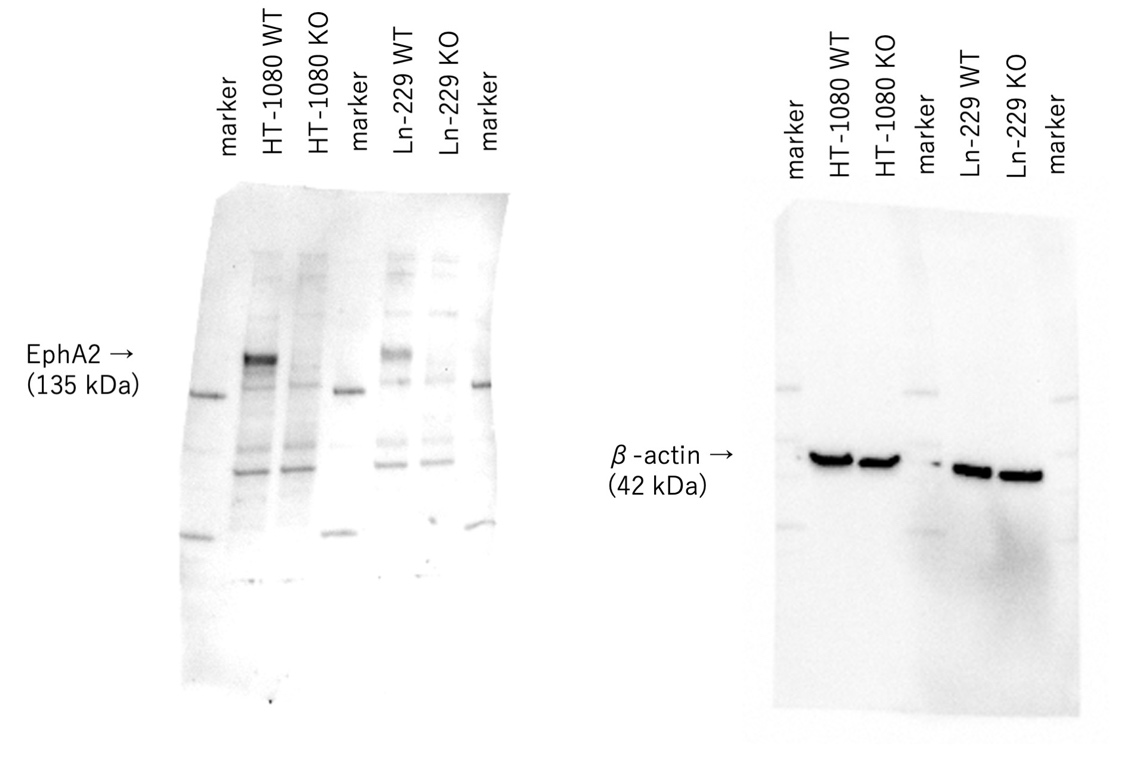
**

**Supplementary Fig. 1.** The full uncropped images of western blotting analysis for EphA2 in HT-1080 WT and HT-1080 EphA2-KO cells.
